# Expansion of the known distribution of Asiatic mouflon (*Ovis orientalis*) in the Late Pleistocene of the Southern Levant

**DOI:** 10.1098/rsos.170409

**Published:** 2017-08-23

**Authors:** Lisa Yeomans, Louise Martin, Tobias Richter

**Affiliations:** 1 Department of Cross-Cultural and Regional Studies, University of Copenhagen, South Campus, Karen Blixens Plads 8, Building 10, 2300 Copenhagen S, Denmark; 2 UCL Institute of Archaeology, 31–34 Gordon Square, London WC1H 0PY, UK

**Keywords:** Epipalaeolithic, Late Pleistocene, Natufian, *Ovis orientalis*, zoogeography

## Abstract

Wild sheep (*Ovis orientalis*) bones recovered from the Natufian site of Shubayqa 1 demonstrate a wider distribution of mouflon in the Late Pleistocene of the Southern Levant than previously known. Early Epipalaeolithic sites are common in the limestone steppe region of eastern Jordan but have yielded only a handful of caprine bones that cannot be identified to species level and few faunal remains from excavated Late Epipalaeolithic sites have been reported. Analysis of animal bone from Shubayqa 1 suggests a significant population of wild sheep could be found concentrated in the basalt desert environment of eastern Jordan during the Late Pleistocene, especially where higher rainfall over the Jebel Druze provided more water. A population of wild sheep was still present in the Pre-Pottery Neolithic A when the nearby site of Shubayqa 6 was occupied. Hunting of diverse, locally available resources including wild sheep at the end of the Pleistocene illustrates the flexible and adaptive exploitation strategies that hunter-forager groups engaged in. This provides further evidence to the increasing body of data showing the creative and opportunistic approach of terminal Pleistocene groups allowing continued occupation even in more marginal environments in a period of environmental change.

## 1. Introduction

The evolution of the genus *Ovis* is complex [1] but the currently accepted view of Old World sheep is that European mouflon (*Ovis orientalis musimon*) and Asiatic mouflon (*Ovis orientalis*) are two distinct subspecies [2,3]. Genetic research has also shown the presence of hybrids between the Asiatic mouflon and the urial (*Ovis vignei*) in Iran indicating that these two are also subspecies [2]. In the literature Asiatic mouflon is occasionally referred to as *Ovis gmelinii* and the urial is still frequently referred to as *Ovis vignei*. The argali (*Ovis ammon*) is a distinct species distributed in mountainous areas of central Asia while the snow sheep (*Ovis nivicola*) is found in northeast Asia [2]. This paper focuses on the Late Pleistocene distribution of Asiatic mouflon (*Ovis orientalis*), hereafter simply referred to as mouflon or wild sheep.

**Figure 1.**
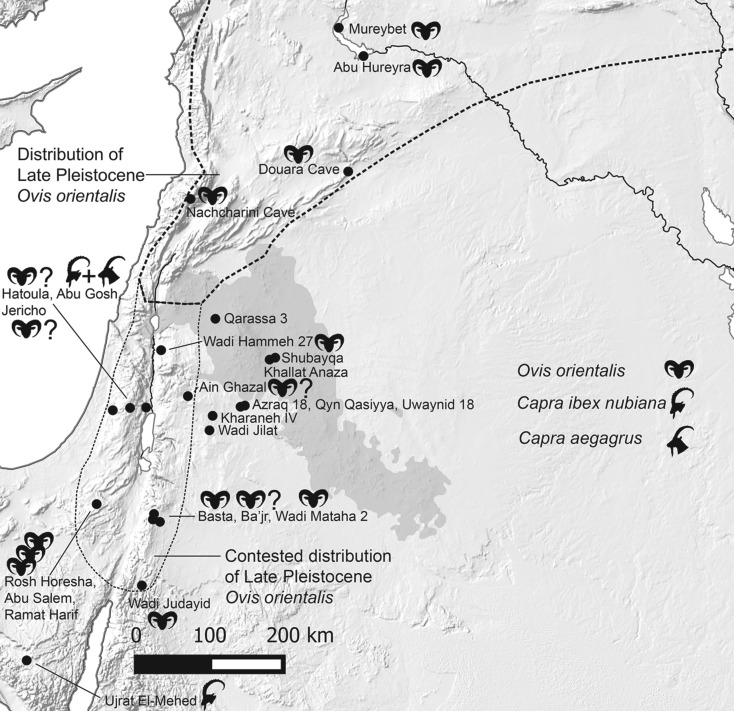
Map of Levant showing the known and contested geographical extent of Late Pleistocene mouflon *Ovis orientalis* prior to the work at Shubayqa. Sites discussed in the text, with symbols indicating the relevant species identified, are also shown.

**Figure 2.**
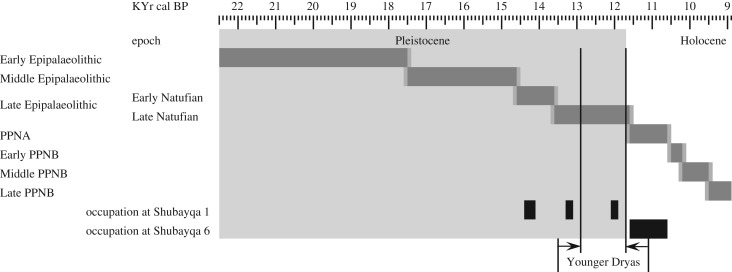
Timeline showing relationship between epochs and archaeological periods and timeframe of occupation at Shubayqa 1 in the Early and Late Natufian (the limited occupation from the final Natufian is off the main mound and not included in the sample of studied faunal remains).

In the Late Pleistocene of southwest Asia mouflon ranged over a geographical area that now forms northern Syria, northern and eastern Iraq, western Iran, and central and eastern Anatolia. They could therefore be found across the area essentially corresponding to that commonly referred to as ‘The Fertile Crescent’ [4,5]. Yet the full ancient geographical distribution of wild sheep is still contested (figure 1) and overlapped with the range of bezoar (*Capra aegagrus*) and Nubian ibex (*Capra ibex nubiana*) making identification of archaeological bones problematic. From Late Pleistocene sites in Anatolia (not covered by figure 1) wild sheep bones have been found at Hallan Çemi Tepesi [6,7] which spans the transition from Late Epipalaeolithic to Neolithic. Figure 2 is a timeline illustrating the Late Pleistocene and Holocene epochs in relation to the archaeological periods discussed. Other evidence of wild sheep in the Early Holocene of Anatolia is plentiful and not reviewed here. The Terminal Pleistocene site of Palegawra Cave in northeast Iraq also contained wild sheep bones among the retrieved faunal remains [8]. In western Iran, sites such as Zawi Chemi Shanidar [9,10] and Warwasi Cave [11] attest to the distribution of mouflon in the Late Pleistocene with further wild sheep bones recovered from Early Holocene sites.

Further south, mouflon have been identified at the Epipalaeolithic sites of Mureybet [12,13], Abu Hureyra [14,15] and also the earlier Palaeolithic site of Douara Cave [16] in the northern Levant. These finds indicate that the species could inhabit relatively open environments beyond the foothills of the Taurus and Zagros mountain ranges. Holocene wild sheep bones have been identified from PPNA (pre-Pottery Neolithic A) deposits at Nachcharini Cave in Lebanon where they form a high proportion of the assemblage [17].

In the southern Levant sheep remains, possibly representing a separate population of Late Pleistocene mouflon, have been identified in the Negev and southern Jordan (table 1) at Rosh Horesha, Abu Salem, Ramat Harif, Wadi Judayid and Wadi Mataha [18,19,23,24]. Wild sheep in these locations may have been part of a refugia group surviving from an earlier wider distribution of the species. However, habitats where wild sheep could be found during the Late Pleistocene included parts of the Jordan Valley based on finds identified at the Natufian site of Wadi Hammeh 27 [22]. A small sample of animal bones were identified from the Natufian site of Qarassa 3 northwest of the Jebel Druze where caprines formed about 10% of the assemblage. Although only a single horncore was identifiable as wild goat [25], it should be noted that horncores may have been transported some distance from where the animals lived or were hunted. Whether mouflon were present in this assemblage remains to be seen. Horwitz & Ducos [26] have argued that wild sheep only survived into the Late Natufian (end of the Late Pleistocene) in the southern Levant and have challenged the reliability of later PPNA finds. While small numbers of wild sheep have been identified in the PPNA assemblages at Hatoula [20] and Jericho [21], it is these bones that later researchers have refuted on the basis of identification and stratigraphic security [26]. Wasse [27] considers the presence of wild sheep at Ain Ghazal in the Jordan highlands unlikely. However, osteometric analysis indicates that some wild sheep were hunted in the vicinity of the site alongside the herding of domestic animals in the PPNB (Pre-Pottery Neolithic B) [28]. Martin & Edwards [28] also raise the possibility of a few wild sheep, represented by larger bones, alongside domestic animals in the LPPNB assemblages from Basta and Baja [29,30] reaffirming Becker's [29] original identification of occasional wild sheep hunting at Basta. While wild sheep do not appear to have inhabited the fertile coastal strip of the Levant [4], their presence in the Jordan Valley is plausible and small numbers may well have survived into the Early Holocene. The distribution of wild sheep in the semi-arid area of eastern Jordan has, until now, been unclear because of a lack of evidence. Wild sheep were clearly capable of living in lower rainfall regions of the Levant as their presence at sites in the Negev demonstrates [19]. A number of Early Epipalaeolithic (*ca* 22 000–17 500 cal BP) sites excavated in the dry steppe and desert environments around the Azraq Basin yielded very occasional caprine bones (see below for more detail) hinting at a wider distribution of either wild sheep or goat in an area beyond the known range of these species in the Late Pleistocene. There has been a dearth of sites excavated in moister environments towards the foothills of the Jebel Druze and the excavation of sites at Shubayqa provide the first large and well-dated faunal assemblages in this region [31–34] creating a new window into past faunal distributions.

**Table 1.** Late Pleistocene and PPNA sites in the Southern Levant where mouflon bones have been identified. (NISP, number of identified specimens; sh, sheep; gt, goat.)

site	period	NISP	sh	gt	sh/gt
Abu Salem [18,19]	Terminal Epipalaeolithic		+		
Hatoula [20]	Natufian levels	2388	?8		
Jericho [21]	PPNA	560	3	10	7
Ramat Harif [19]	Terminal Epipalaeolithic		+		
Rosh Horesha [18,19]	Natufian		+		
Wadi Hammeh 27 [22]	Early Natufian	1367	14	6	101
Wadi Judayid [23]	Early Natufian	214	28	24	76
Wadi Mataha 2 [24]	Early Natufian	257	2		143
Wadi Mataha 2 [24]	Late Natufian	627	3		183

## 2. Shubayqa

Shubayqa is located in the Harrat ash-Shaam (Black Desert) in northeast Jordan (figure 1). A number of prehistoric sites border the Qa’ Shubayqa which is a 12 km^2^ shallow basin fed by a series of wadis. The Wadi Rajil is the most significant of these and directs rain water from the Jebel Druze into the playa. Today localized flooding occurs after the winter rains between October/November and March/April.

Shubayqa 1 and Shubayqa 6 date to the Natufian and PPNA respectively and are both located on the northern side of the Qa’ Shubayqa approximately 900 m apart. The stratigraphic sequence of Shubayqa 1 is divided into a number of phases that represent occupation in the Early Natufian and, after a hiatus, further occupation in the Late Natufian [31,32]. The sequence is dated by 22 Accelerator Mass Spectrometry (AMS) dates with 17 from the Early Natufian sequence (approx. 14 400–14 100 cal BP) and five dating the Late Natufian (approx. 13 100–13 300 cal BP). A single date (approx. 12 100–11 900 cal BP) from a small test trench suggests that limited occupation continued into the Younger Dryas but minimal animal bone was recovered from this area. Excavations continue at Shubayqa 6 but several PPNA structures have been excavated and, on the basis of a test trench, the sequence appears to extend back to the Late Natufian [34–36]. Five AMS dates span the timeframe 12 370–10 590 cal BP (68.2% probability) placing the main sequence within the PPNA although the lithic assemblage suggests reoccupation in the EPPNB.

Excavations at Shubayqa 1 are complete and analysis of faunal remains has allowed discussion of temporal shifts in hunting practices, seasonal variation in resource exploitation and an insight into changing environmental conditions [37]. During the Natufian the most commonly exploited animals were gazelle, and wintering and passage migrant birds [37]. In total, more than 17 000 animal bones (NISP; number of identified specimens) have been identified from Shubayqa 1, with birds forming over a third and gazelle representing more than 40% of the total. Other species represented include onager (approx. 1%), wild cattle (less than 1%), fox (approx. 1%), hare (approx. 6%), tortoise (less than 1%) and caprines (approx. 4%) which are the subject of this paper. Analysis of the extensive faunal assemblage from Shubayqa 6 is ongoing and about 600 bones have been identified at this stage. Preliminary results suggest that birds were of less importance than in the Natufian (approx. 16%), gazelle constitute more than 50% of the assemblage and hare and fox are more frequent than at Shubayqa 1 (approx. 12% and approx. 5% respectively). Of particular relevance here is that initial results suggest that caprines were as frequent in the PPNA assemblage (approx. 5%) as in the Natufian assemblage from Shubayqa 1.

## 3. Identification of the caprine bones from Shubayqa

Potential candidate species for the caprine bones from Shubayqa are bezoar (*Capra aegagrus*), Nubian ibex (*Capra ibex nubiana*) and mouflon (*Ovis orientalis*) with the bones possibly deriving from more than one species. These species are all within the Caprini tribe of the Caprinae subfamily. Separating the bones of sheep and goats in general has long been challenging to zooarchaeologists because of the similar osteology of the two genera. This is compounded further when dealing with highly fragmentary bones such as those from Shubayqa. Without wishing to pre-empt our zooarchaeological identifications, we first consider which species are likely to have been in the vicinity of Shubayqa given what we know of past caprine distributions, their habitat preferences and the Late Pleistocene environment around Shubayqa. Recent and historical wild caprine distributions cannot be used to inform on this question since wildlife in the region has been heavily impacted by millennia of over-hunting, livestock herding and ecological change.

During the Late Pleistocene the Southern Levantine environment was considerably wetter than today, which is evidenced at Shubayqa by the high occurrence of wetland birds as well as the remains of wetland plants. The Nubian ibex is better adapted to arid environments than the bezoar goat, with southern Jordan considered towards the northern-most extent of the range of ibex today. Ibex prefer rugged, very steep and mountainous terrain which they can scramble up with outstanding ease to evade predators [38,39]. Such a landscape does not typify the environment in the vicinity of Shubayqa. It is likely, however, that ibex did inhabit parts of eastern Jordan's Black desert in the Holocene given the common occurrence of ibex in rock art [40]. The areas surveyed by Rollefson and colleagues centred on the high mesas of the southeast basalt desert, which seem well-suited to ibex. Rock art is notoriously difficult to date but scholars suggest an early Holocene date at the earliest for these animal engravings, and whether they represent common wildlife, or rare occurrences is impossible to tell [40]. Tchernov [41] has argued that arid adapted faunas (such as oryx and Arabian gazelle) shifted northwards when Holocene environmental conditions took hold and it is far from certain that any of the Saharo-Arabian mammals such as Nubian ibex were part of the Late Pleistocene landscapes of the southern Levant. The palaeoenvironments and topography of Natufian Shubayqa appear far better suited to either the bezoar or mouflon.

Turning to the caprine bones themselves, we can consider metrical and morphological methods of identification as well as comparing the sizes of the bones to those from other sites where ibex, bezoar and mouflon have been identified. The last of these approaches can be more problematic due to variation in the size of animals in different populations because of environmental factors. In the following sections we work through these different methods of identification starting with the techniques that are considered more reliable and less subjective.

### 3.1. Metrical analysis of morphology

**Figure 3.**
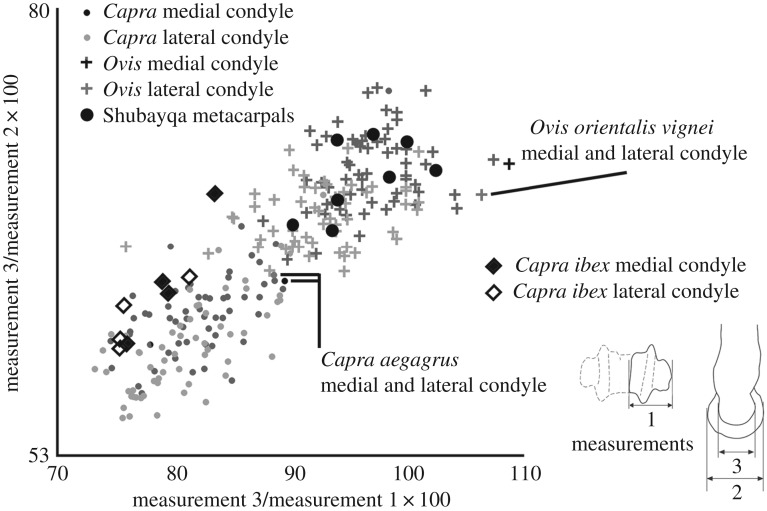
Differentiation of sheep and goat based on measurements taken on the distal metacarpal following Salvagno & Albarella [47] with additional measurements from wild sheep, bezoar and ibex from the Zoological Museum, University of Copenhagen.

The metacarpal is one of the most useful skeletal elements for separating sheep and goat on the basis of the relative proportions of parts of the bones. No complete metacarpals were recovered so it is not possible to compare the length (GL) to the breadth of distal end (Bd) as suggested by Boessneck [42,43]. There are, however, methods for exploring the proportional differences of the distal metacarpal of sheep and goats [44–46]. A refinement to this approach of osteometric identification of sheep and goat metacarpals [47] was also applied to the Shubayqa caprine metacarpal remains. Using this method the Shubayqa specimens (figure 3) consistently identify as sheep regardless of whether the points are compared to the graphs for the medial or lateral condyle. This is important since a number of the metacarpals from Shubayqa are broken leaving only one condyle which may have come from either the medial or lateral side of the bone.

Separation of the metatarsals is more problematic since there is degree of overlap between sheep and goats in the measurements taken on the distal articulation. However, if the measurements are plotted on the graphs of Salvagno & Albarella [47], the points fall in the areas of the graph dominated by sheep or exclusively sheep. These metrics are provided in electronic supplementary material but not presented graphically.

**Figure 4.**
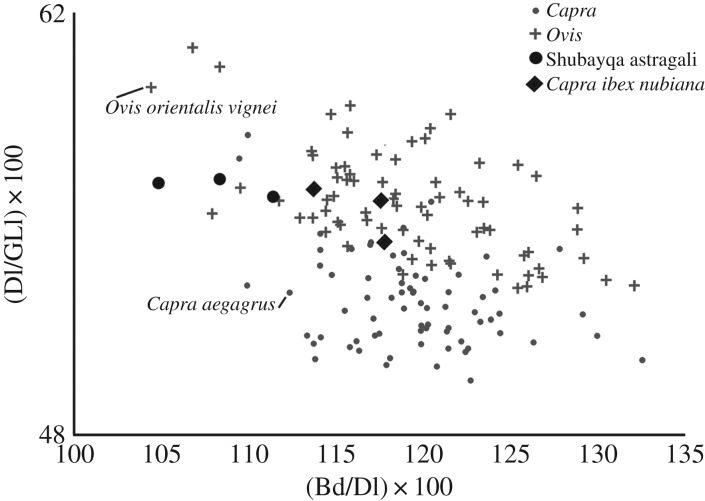
Metrical identification of caprine astragali following Salvangno & Albarella [47] and Davis [48] with additional measurements from wild sheep, bezoar and ibex from the Zoological Museum, University of Copenhagen.

Salvagno & Albarella [47] as well as Davis [48] presented metrical methods for separating the astragali of sheep and goats using standard measurements and again the measurements of Shubayqa bones fall into the portion of the graphs dominated by sheep (figure 4). However, the evidence is less clear cut than with the metacarpals especially when measurements taken on wild sheep, bezoar and ibex astragali from the modern reference collection held at the Zoological Museum, University of Copenhagen are included for comparison. The only other element including enough metrical data to compare to the methods described in Salvagno & Albarella [47] is the third phalanx. Here the points fall in the area of the graph where sheep and goat overlap.

### 3.2. Morphological identification

**Figure 5.**
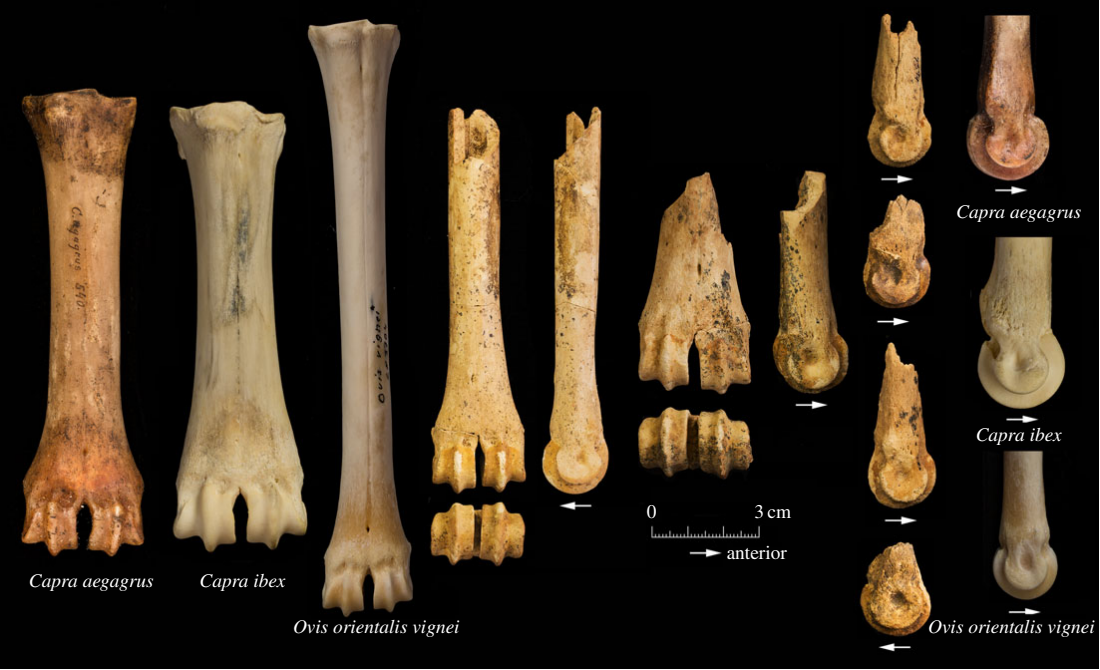
Metacarpal fragments from Shubayqa compared to those of wild sheep, bezoar and ibex from the Zoological Museum, University of Copenhagen.

Morphological characteristics for identification of sheep and goat have been summarized by Zeder & Lapham [45] mainly for skeletal elements that provide fusion data. Rowley-Conwy [46] also reviewed methods for separating metapodia of sheep and goats. Publications detailing methods of separating bones based on additional elements including the third phalanx and some carpal bones were also used [42,43]. The metacarpal, as well as being metrically differentiable, is also clearly morphologically different between sheep and goats. In some respects the two methods are related since those characteristics that can be represented metrically are also visible morphologically. Additional criteria, such as whether the verticillus of the medial and lateral condyles are parallel, or the pronounced notches in the distal shaft immediately proximal by the verticillis, cannot be measured. These characteristics on the caprine metacarpals from Shubayqa are typical of sheep (figure 5) and, where the length compared to breadth is visible on the more complete metacarpals, there can be little doubt that the bone derived from a sheep.

**Figure 6.**
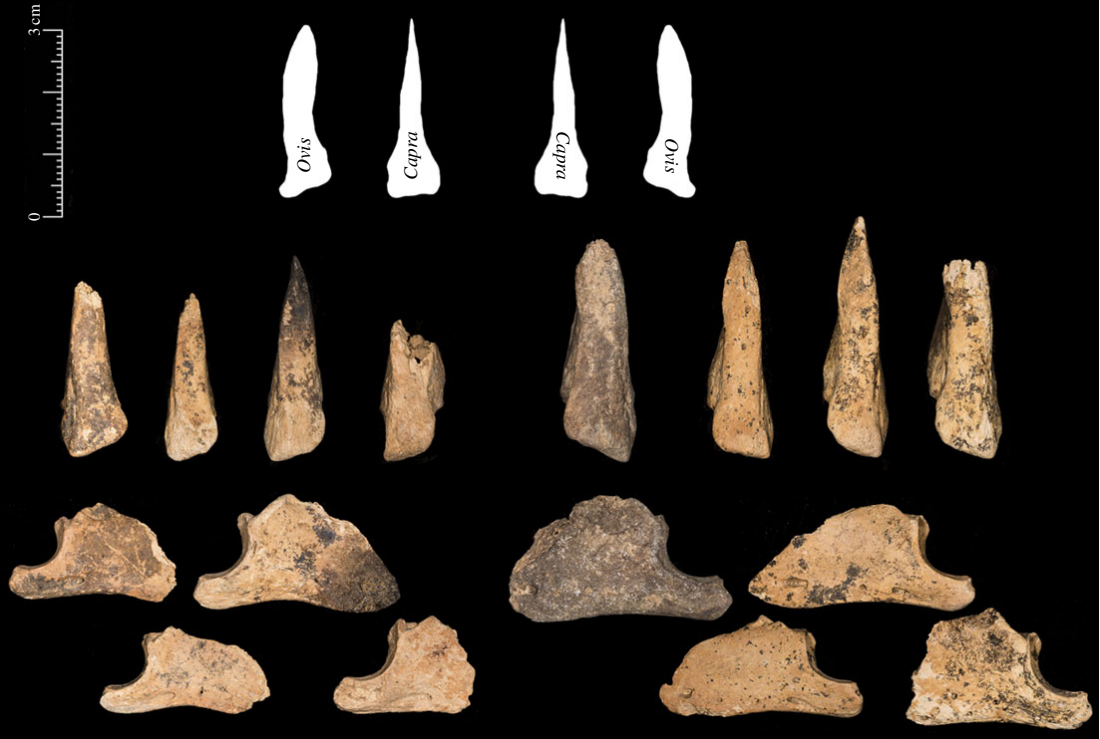
Eight caprine third phalanges from Shubayqa 1; top line shows ventral outline of typical sheep and goat third phalanges [42,43], middle row shows the ventral view of the bones identified to mouflon and lower row shows lateral aspects of the same bones.

The third phalanx, which is often easier than many elements to accurately classify to sheep or goat [42,43], was recovered in some numbers. As seen in figure 6 these bones, when viewed from the ventral side, display the curved outline typical of sheep and not the sharp triangular shape found in goats. Viewed from the lateral aspect, the large size of the process extensorius can be seen in cases when it has not been damaged.

**Figure 7.**
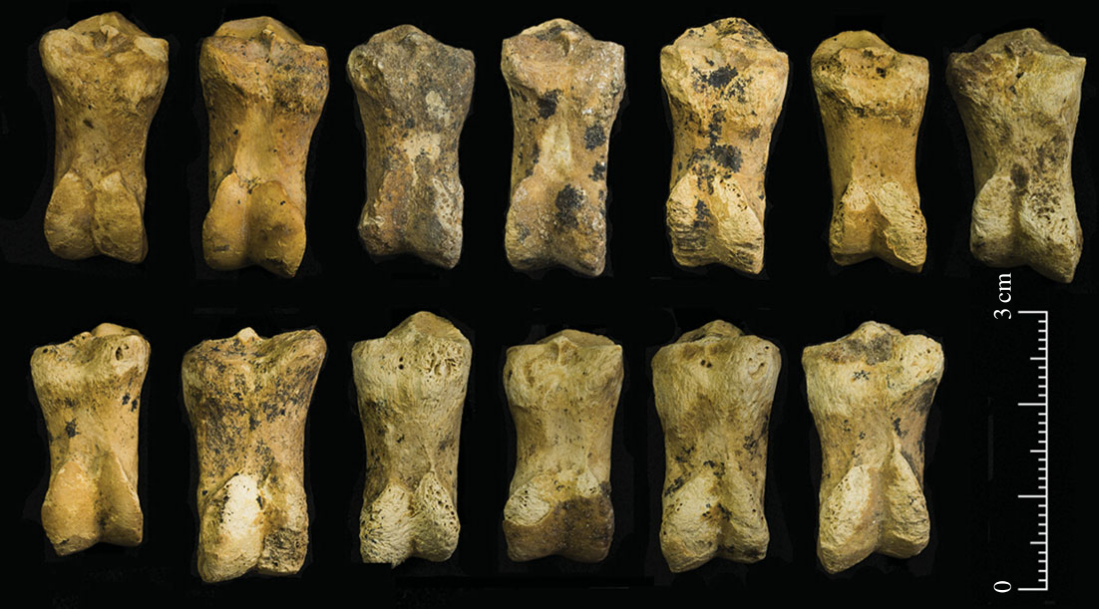
Thirteen second phalanges from Shubayqa 1; top row are left lateral or right medial bones and the lower row are right lateral or left medial bones.

Other elements commonly found include the second phalanx (figure 7). These bones tend to conform to sheep with the two halves of the distal articulation showing less asymmetry than expected in goats. On the posterior surface, the peripheral side of the articulation does not extend markedly as in goat phalanges [42,43].When viewed from the distal end, it is also clear that there is not a substantial size difference between the axial and peripheral sides of the condyle. Boessneck [42,43] describes these characteristics as typical of sheep although identification is not straightforward.

**Figure 8.**
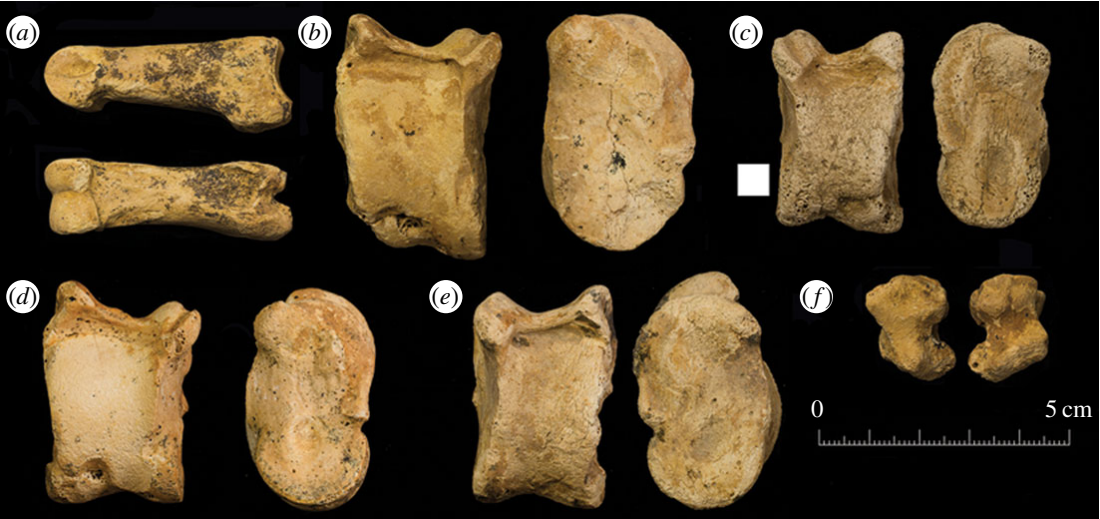
Selection of other bones from Shubayqa; (*a*) first phalanx showing axial and posterior aspects, (*b*–*e*) astragalus viewed from the plantar and medial sides and (*f*) intermediate carpal showing proximal and distal aspects.

First phalanges can be more problematic to identify but those present in the Shubayqa assemblage (figure 8*a*) are typical for sheep based on the shape of the proximal end inversely mirroring the different morphologies of sheep and goat distal metapodia. Additionally the posterior side of the shaft is often fairly concave in form without pronounced points for ligament attachment on the lateral margins. At the distal end the best distinguishing criterion is the lack of a pronounced division between the axial and peripheral sides of the articulation where it extends onto the ventral surface [42,43]. Other elements morphologically similar to sheep from the Shubayqa assemblage include some complete astragali (figure 8*b–e*). Among other characteristics, these bones show the strong ridge on the medial part of the articulation on the plantar side and a strong lobe when viewed from the medial side. As high bone fragmentation renders many elements unidentifiable, separation was attempted on carpals as these small dense bones tended to survive complete. Zeder & Lapham [45] mention that intermediate and ulnar carpals seem to offer the most reliable means for separating sheep from goat. As a preliminary test, the criteria described for these elements by Boessneck *et al*. [42] were applied to modern reference sheep and goat skeletons at the Zoological Museum, University of Copenhagen. This found that only intermediate carpals offered consistently reliable criteria since the facet between intermediate and ulnar carpals was variable. The pronounced angle on intermediate carpal illustrated by Boessneck *et al*. [42] in his figures 39(a) and 41(a) clearly separated bones of the sheep and goat (figure 8*f*).

### 3.3. Metrical comparison to other sites

**Figure 9.**
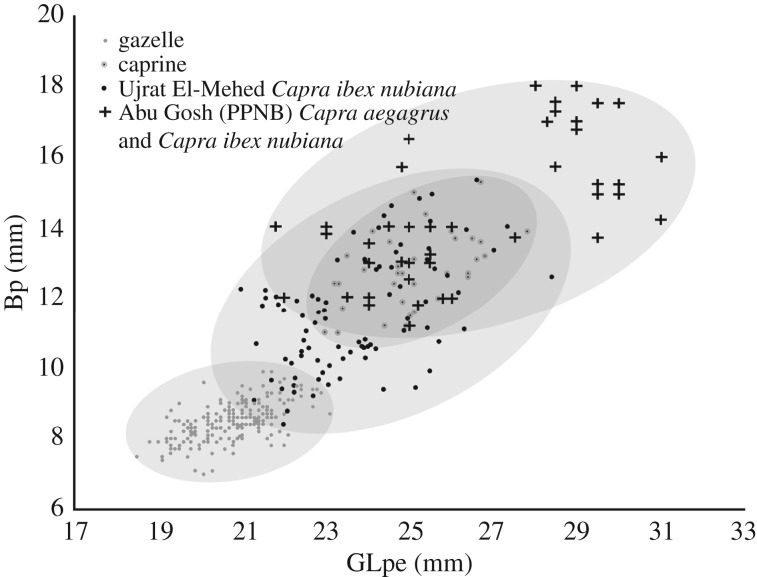
Comparison of measurements taken on caprine second phalanges showing the range of sizes present in different assemblages.

Another potential method for identifying the caprine remains from Shubayqa is to compare their size to identified bones from other sites. There are caveats to this approach as it assumes minimal variation in a species size either temporally or geographically. Within the Shubayqa assemblage the second phalanx was the only element complete enough to take a significant number of measurements. Figure 9 shows the distribution of these measurements in relation to the large assemblage of ibex bones from Ujrat El-Mehed [49], an assemblage from Abu Gosh of bezoar with some ibex also present on the basis of DNA analysis [50,51], as well as the gazelle from Shubayqa simply to show the size variation between the caprines and gazelle at the site. There are very few wild sheep bone assemblages to compare measurements on this specific bone. The range of sizes of the caprine phalanges from Shubayqa is smaller than that of the ibex from Ujrat El-Mehed. This could be related to a strong degree of sexual dimorphism in ibex with published indices of sexual dimorphism given as 2.36 for *Capra ibex nubiana*, 1.59 for *Ovis orientalis* and 1.30 for *Capra aegagrus* [52]. This suggests that the bones from Shubayqa are from one of the species with a lower degree of sexual dimorphism. Of course one could argue that only large male ibex were hunted but this seems unlikely. The range of sizes of the second phalanges from Abu Gosh is wide; this is difficult to interpret but may relate to the presence of both bezoar and ibex in the assemblage. Although there is no sizable assemblage of mouflon second phalanges for comparison, figure 9 is useful to show the range of sizes compared to assemblages of bezoar and ibex.

**Figure 10.**
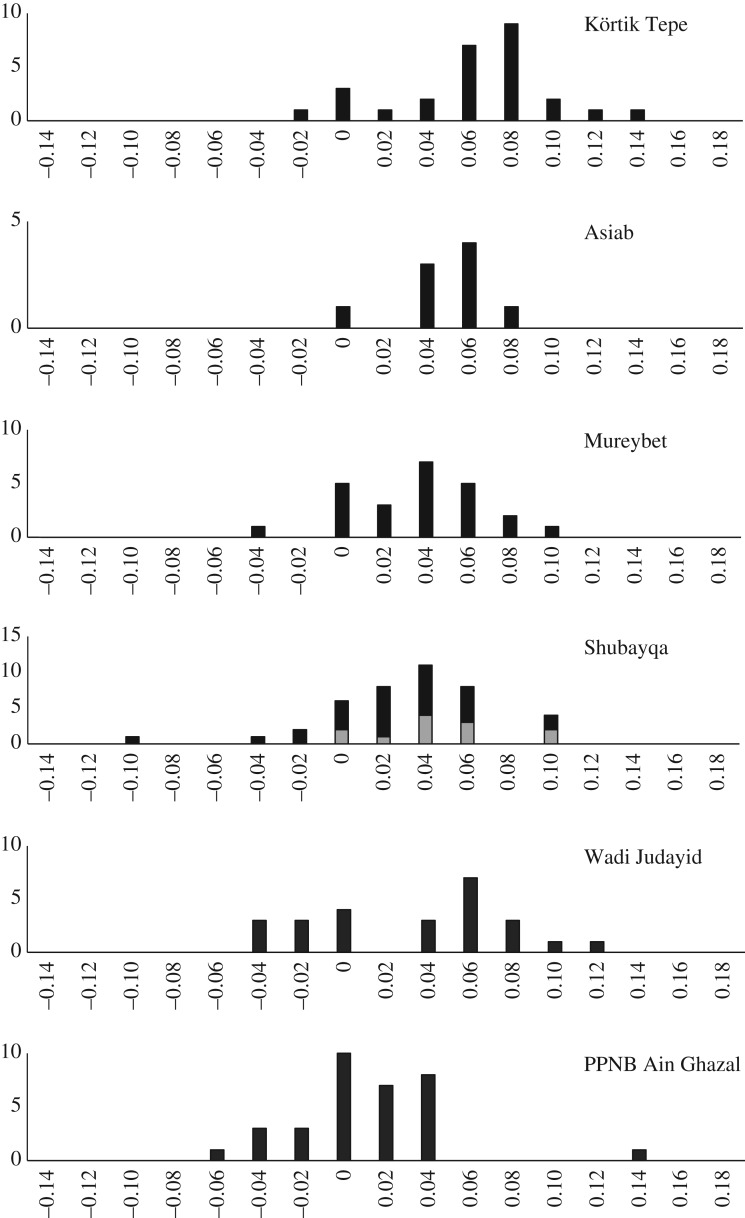
Size comparison of wild sheep from sites from southwest Asia across a range of environments. Size calculated as log standard index after Uerpmann & Uerpmann [53], after Martin & Edwards [28] incorporating data from Bökönyi [54] and Arbuckle & Özkaya [55]. For Shubayqa grey bars represent identified sheep and open bars are sheep/goat; given that there is no evidence for goat in the assemblage these probably also derive from sheep. PPNB Ain Ghazal included showing the difference to domestic sheep populations that have undergone size reduction [28].

Using the log standard index (LSI) method of Uerpmann & Uerpmann [53] allows comparison of measurements from different elements combined (figure 10) and must be resorted to when there is a lack of data from a single element. Figure 10 provides an indication that the caprines can be considered as similar in size to the wild sheep in the region based on finds of wild sheep remains from Wadi Judayid [23], Mureybet [12,13], Asiab [10,54] and Körtik Tepe [55]. These sites provide samples of wild sheep across a wide geographical range from southern Jordan, the Euphrates Valley, Zagros foothills and eastern Anatolia. At sites towards in the southeastern part of this range the sheep are marginally smaller probably reflecting Bergman's rule and compares well to the geographical size trajectory noted for sheep and goats in general [10,56]. Broadly, however, the sizes of the caprines from Shubayqa are similar to that of wild sheep at other sites. For comparison figure 10 includes domestic sheep from PPNB Ain Ghazal to illustrate the difference between wild sheep and those that have undergone size reduction after generations of breeding.

### 3.4. Summary

Taken together, the evidence presented above leads us to the conclusion that the caprine bones from Shubayqa are mouflon. This interpretation has been reached on the basis of ecological evidence, metrical analysis of the shape of various elements but especially the metacarpals, and morphological criteria which can be more subjective. The narrow range of size of the second phalanges seems to suggest that only one species is represented and, while there is substantial overlap between the sizes of the three candidate species, the sample of bones are not dissimilar to other reported wild sheep in the region. In summary the zooarchaeological analysis demonstrates the presence of sheep within the securely dated deposits of Shubayqa 1 during the Natufian. The presence of goat (either bezoar or ibex) cannot be entirely ruled out as heavy fragmentation has limited the number of bones that can be identified to species level. However, given that 143 bones have been identified as sheep with none as goat, it seems probable that only sheep were present. In total 510 bones were classified as sheep/goat. Together caprines formed 6.3% of the mammal (hare-sized or larger) faunal assemblage and therefore were an important constituent of the hunted prey. Despite the limited sample of bones currently studied from Shubayqa 6, 29 bones have been identified as sheep/goat and a further four are sheep indicating the sheep population continued in the Black Desert during the Younger Dryas or repopulated the area after this climatic event.

## 4. Wild sheep in the Black Desert

There is an absence of data on the distribution of sheep prior to the Epipalaeolithic in the eastern desert since very few faunal assemblages have been studied from the Upper Palaeolithic outside of the Mediterranean zone [56]. Table 2 summarizes data for identified caprines alongside the most common taxon, gazelle, from Upper Palaeolithic to PPNA sites in eastern Jordan. Caprines have been identified in very low numbers at some Epipalaeolithic sites in the limestone steppe and close to the Azraq Oasis, but none of the bones could be identified further to sheep or goat [58,59,61]. A very limited sample of faunal remains was identified from Khallat Anaza of Late Epipalaeolithic date and sheep/goat bones were well represented. Identification to species level was difficult and only two bones tentatively assigned to goat [60]. Khallat Anaza lies by an outcrop of bedrock basalt at a bend in the Wadi Rajil southwest and upstream of Shubayqa [62]. A series of pools cut into the basalt by the wadi would have provided a water source well beyond the rainy season. A similar site, Mugharet el-Jawa is located a further 8 km upstream to the northwest and is located in a similar environmental setting as Khallat Anaza [62]. These sites, along with similar ones along the route of the Wadi Rajil, would have provided attractive, well-watered locations for people and animals traversing the basalt desert. The course of the Wadi Rajil curves around the Jawa basalt flow which redirected the original course of the seasonal waters [63]. At Shubayqa, the Wadi Salma feeds into the Wadi Rajil which then continues in a meandering southerly direction along the eastern side of the lava flow, eventually turning towards the Azraq Oasis. Human mobility in the Late Pleistocene around this semi-arid landscape must have been facilitated by following the courses of these natural thoroughfares and hunting parties presumably targeted prey attracted to the water.

**Table 2.** Number of identified specimens of sheep, goat, sheep/goat and gazelle bones (sh/gt/gaz excluded) from Epipalaeolithic sites in eastern Jordan.

			NISP			
site	location	date	gaz	sh	gt	sh/gt
Uwaynid 18 [5]	Limestone steppe	Late Upper Palaeolithic to initial Epipalaeolithic	431	0	0	1
Wadi Jilat 6 [58]	Limestone steppe	Late Upper Palaeolithic to Early Epipalaeolithic	1983	0	0	0
Kharaneh IV [58]	Limestone steppe	Early Epipalaeolithic	9885	0	0	0
Ayn Qasiyya [59]	Azraq Oasis	Early Epipalaeolithic	3737	0	0	3
Wadi Jilat 22 [5]	Limestone steppe	Mid to early Late Epipalaeolithic	861	0	0	13
Khallat Anaza [60]	Basalt desert	Late Epipalaeolithic	7	0	2?	17
Azraq 18 [5]	Azraq Oasis	Late Epipalaeolithic	58	0	0	0
Shubayqa 1	Basalt desert	Early Natufian	2981	26	0	92
Shubayqa 1	Basalt desert	Late Natufian	4128	117	0	418
Shubayqa 6	Basalt desert	PPNA	316	4	0	29

**Figure 11.**
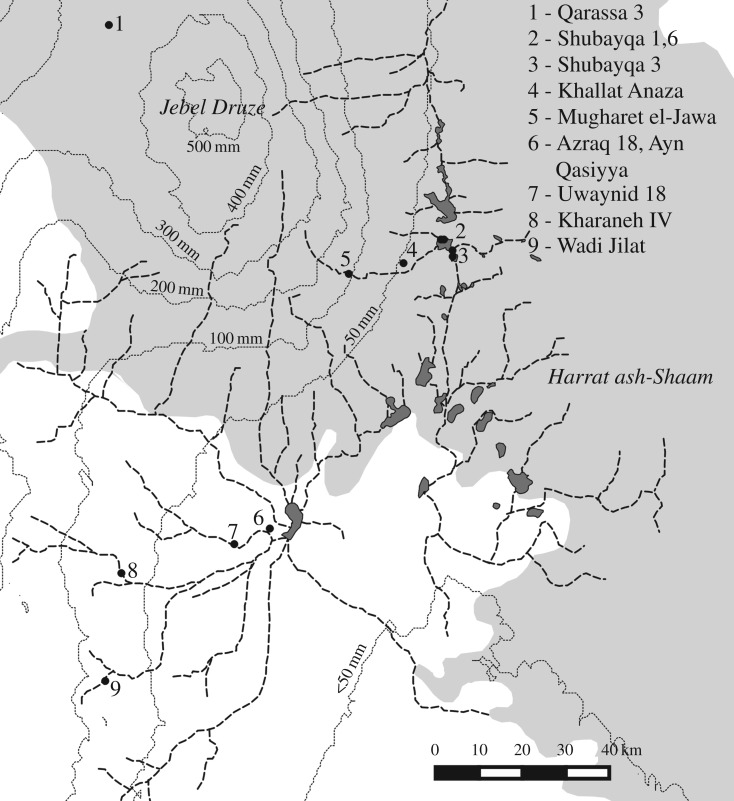
Eastern desert area with location of sites discussed with current rainfall, major wadi systems and the extent of the basalt desert shown.

The relatively high frequency of sheep/goat at Khallat Anaza, given the caveat of small sample size, hints that caprines were more common further upstream of Shubayqa closer to the Jebel Druze. This is not surprising given that the environment towards the foothills of the Jebel Druze would have received higher rainfall. Even today, in a much changed environment, the southern slopes of the Jebel Druze receive at least 100 mm more rainfall than Shubayqa. Wild sheep would therefore have found favourable habitats towards the higher ground of the Jebel Druze, and the occasional caprine bones present at sites in the limestone steppe may have resulted from hunting trips into the basalt desert, or instances of sheep forays into the steppe during winters. Figure 11 shows the location of sites in the eastern desert, current rainfall, major wadis and the extent of the basalt boulderlands. Also indicated are other Late Epipalaeolithic sites identified in the region suggesting a network of locations along the lines of the wadis which hunter-gatherer groups used as main routes across the landscape. Many of the sites identified are close to the Syrian border and unfortunately cannot be investigated more intensively at this time, but it can be hypothesized that in future these sites may demonstrate sheep to be a more common component of the local wildlife since they are closer to the Jebel Druze where the environment and vegetation was lusher. It is likely that Shubayqa was towards the southerly limit of the preferred habitat of wild sheep, which might explain the difference between their significant presence at Shubayqa 1 compared to their paucity at other Epipalaeolithic sites across the limestone steppe.

The Late Pleistocene is a period of significant climatic variation with the Younger Dryas notable as a cooler and drier phase after the Bølling-Allerød interstadial. The sample of sheep bones from the Early and Late Natufian phases predates the Younger Dryas and the sample from Shubayqa 6 postdates this climatic period. Across the broad Late Pleistocene to Early Holocene transition the frequency of sheep remained relatively stable but there is a notable increase in sheep frequency during the Late Natufian. Analysis of the entire faunal assemblage from Shubayqa 1 [64] suggests that climatic shifts had already begun influencing the prey availability in the Late Natufian resulting in some hunting activities taking place further away from Shubayqa and upstream towards the Jebel Druze. This may explain the short-lived increase in the frequency of sheep as populations increased mobility to seek additional prey before mobility levels declined in the PPNA. The influence of climatic shifts needs further consideration once additional data from the PPNA assemblage is collated but it seems that the wild sheep population was largely resilient and could be hunted in the Black Desert across this period of climatic instability either remaining in the environment throughout the Younger Dryas or quickly repopulating the Black Desert after the climatic downturn came to a close.

## 5. Conclusion

This paper has documented the presence of mouflon in the Late Pleistocene and Early Holocene of the Black Desert on the basis of morphological and metrical analysis of the faunal remains from Natufian deposits at Shubayqa 1 and PPNA deposits at Shubayqa 6. Bone collagen was not preserved well and ZooMS (Zooarchaeology by Mass Spectrometry) analysis was unsuccessfully attempted to provide biomolecular confirmation of the morphometric results presented here. Ancient DNA analyses are unlikely to yield results for the same reasons. We are confident that the presence of mouflon at Shubayqa is unrelated to later contamination because wild sheep bones are present throughout the stratigraphy, including the earliest site occupation phases sealed below basalt slabs from the floors of the Natufian structures. Caprine body-part representation, although heavily skewed towards elements that survive carcass-processing for marrow and grease, suggests that sheep were hunted in the vicinity of the site. There is no evidence that sheep carcasses were processed at kill-sites with only major meat-bearing elements transported to Shubayqa 1; therefore we argue that hunting would have occurred relatively close-by. Furthermore, the carcasses of mouflon were treated in the same manner as those of other prey species; the NISP from different elements identified as gazelle or caprine (see electronic supplementary material) do not differ (*χ*^2 ^= 1.75 × 10^−38^).

Although mouflon were present in the Black Desert landscape around Shubayqa, they were clearly not hunted as often as gazelle and the relative frequency of the two species (92% gazelle compared to 8% sheep) probably reflects their relative abundance in the landscape. The local environment must have provided a mosaic of resources offering both browsers and grazers sufficient nutrients. Mouflon are non-territorial and not known to have been migratory, although there are few studies that document the behaviour of remaining wild sheep populations and those that do are based on small surviving refugia groups. We can assume that wild sheep would have inhabited the local environment year-round and formed an important resource for the human population to target for food. Most significantly, however, the presence of the substantial number of bones identified as mouflon extends the known range of wild sheep [4,65]. We cannot rely on broad-scale maps showing ancient wild animal distributions as neat lines. Local ecological settings that could have provided the vegetation and water availability to provide a habitat for a species need to be considered. We also know that the mouflon still inhabited the local environment in the PPNA after the Younger Dryas and therefore coped with this climatic event. Wild sheep offered the Natufian and PPNA populations one of a myriad of resources that could be exploited during the Late Pleistocene even in this more marginal environment beyond the Mediterranean zone. Despite the influences of climate on the resources presented to these hunter-foragers, their subsistence strategies were flexible and could cope by shifting focus, and variation in the extent to which mouflon were hunted is just one of the ways that this is reflected in the archaeological record.

## Supplementary Material

Measurements taken on distal condyle of sheep metapodials from Shubayqa

## Supplementary Material

Measurements taken on sheep and sheep or goat bones

## Supplementary Material

Number of identified specimens according to element for gazelle and caprines

## Data accessibility

The data supporting this article are available in the electronic supplementary material.
